# Suture restriction of the temporal bone as a risk factor for acute otitis media in children: cohort study

**DOI:** 10.1186/1471-2431-12-181

**Published:** 2012-11-20

**Authors:** Chantal Morin, Dominique Dorion, Jean-Marie Moutquin, Mélanie Levasseur

**Affiliations:** 1 School of Rehabilitation, Faculty of Medicine and Health Sciences, University of Sherbrooke, 3001 12e Avenue nord, Sherbrooke, QC J1H 5N4, Canada; 2 Division of Otolaryngology-Head and Neck Surgery, Faculty of Medicine and Health Sciences, University of Sherbrooke, 3001 12e Avenue nord, Sherbrooke, QC, J1H 5N4, Canada; 3 Department of Obstetrics-Gynecology, Faculty of Medicine and Health Sciences, University of Sherbrooke, 3001 12e Avenue nord, Sherbrooke, QC, J1H 5N4, Canada; 4 National Institute of Excellence in Health and Social Services, 2021, Avenue Union, bureau 10.082, Montréal, QC, H3A 2S9, Canada

**Keywords:** Acute otitis media, Temporal bone, Children, Eustachian tube, Risk factor, Cohort, Cranial suture, Osteopathy

## Abstract

**Background:**

Eustachian tube (ET) dysfunction plays an important role in the pathogenesis of acute otitis media (AOM). Unfortunately, there is a lack of knowledge about the exact role of the ET’s bony support, the temporal bone, on occurrence of AOM. This study investigates whether severe suture restriction of the temporal bone is a risk factor for development of AOM in young children.

**Methods:**

Using a prospective cohort design, 64 children aged 6 to 18 months without prior history of AOM were followed during the cold season (September 2009 to April 2010). Temporal bone status (categorized as with or without severe suture restriction) was evaluated using palpation and a cranial bone mobility test. Information about potential baseline confounders and risk factors for AOM (gender, age, birth weight, gestational age, use of pacifier, daycare attendance, presence of siblings, low socioeconomic status, breastfeeding ≥ 6 months, parental smoking and history of upper respiratory tract infection) were also collected. Occurrence of AOM diagnosed by physicians blinded to temporal bone status was the main outcome. Data were analyzed using hierarchical linear and nonlinear (multilevel) models.

**Results:**

Severe suture restriction of the temporal bone was identified in 23 children (35.9%). At least one AOM episode was diagnosed in 14 (48.3%) of the ears associated with temporal bones previously identified as having severe suture restriction and in 28 (28.3%) of those without severe suture restriction. Higher risk for AOM was explained by severe suture restriction of the temporal bone (adjusted relative risk (RR), 2.26, 95% CI 1.43 to 2.91, p<.01), pacifier use (RR, 2.59, 95% CI 1.51 to 3.22, p<.01) and younger age (RR, 0.22, 95% CI 0.10 to 0.52, p=.001).

**Conclusions:**

The study results indicate that severe suture restriction of the temporal bone is a risk factor for AOM in young children. Subsequent intervention studies are needed to determine if this mechanical risk factor can be modified in young children.

## Background

Acute otitis media (AOM) is one of the most common childhood infections [1], with important societal and individual consequences. From 1995 to 2003, AOM incidence rates in children under 2 years of age increased by 46%, while antibiotic prescription rates went up by 45% [2]. This situation not only contributes to antibiotic resistance but also creates a significant financial and social burden. AOM generates direct and indirect costs, including medical visits, antibiotics used, parental work time lost and decreased quality life of both child and parents [3,4]. Consequently, the prevention of AOM through reduction of risk factors is a high priority in public health [5] and an important research topic [6,7].

**Figure 1:**
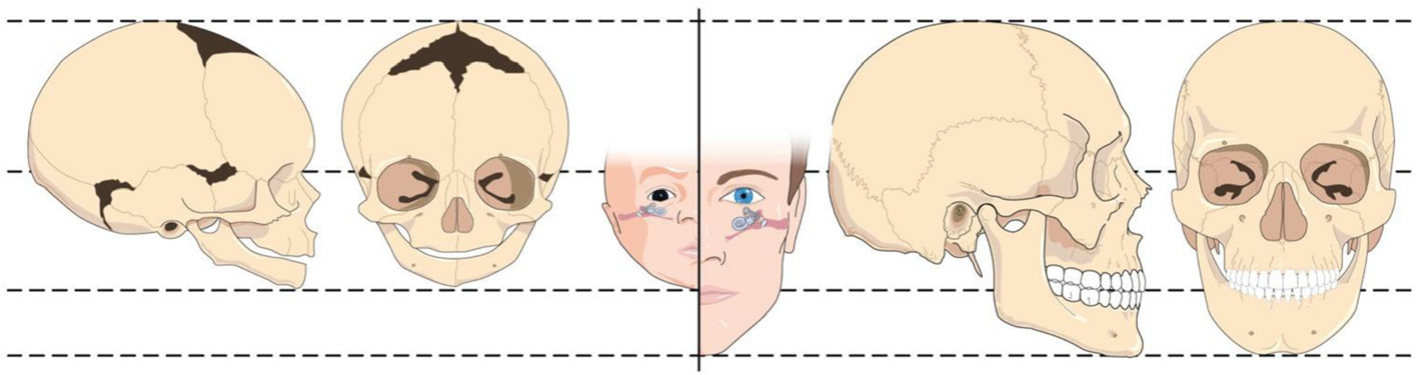
**Craniofacial growth and development affecting the Eustachian tube in children.** Growth and development of the occipital, the sphenoid, the temporal bone and the inferior maxilla move the ET vertically (10 degrees to 45 degrees) and lengthen it. Figure also shows the membranous tissues around the temporal bone in young children where restriction of the suture may happen.

For the last 15 years, efforts using observational studies, have focused on identifying and better understanding risk factors for AOM. Among them, the use of pacifier and attendance at a daycare center have been categorized as modifiable risk factors [8], but most of the other risk factors such as absence of breastfeeding, parental smoking or presence of siblings remain, at best hard – and most of the time impossible – to modify. Eustachian tube (ET) dysfunction has been demonstrated to be the most important risk factor in the pathogenesis of AOM [9] and has traditionally been included with these hard-to-modify factors. Studying the structural properties that govern ET function is necessary to understand which components might be a target for treatment [10]. It is already known that anatomical (short, flaccid and horizontal) and physiological (dynamic opening and mucociliary function) characteristics of the ET contribute to its vulnerability in the young child [11]. During the first five or six years of life, however, anatomical development of the ET reduces its susceptibility to AOM. Specifically, the ET axis moves vertically and its dynamic opening by the tensor veli palatini muscle improves [12]. These anatomical and physiological changes are determined by craniofacial growth and development [13,14] (Figure 1). This growth of the skull base, responsible for much of the cranial lengthening, is dependent on the most important basicranium synchondroses along with the occiput, sphenoid and temporal bones in between. DiFrancesco and colleagues (2008) have investigated the influence of craniofacial morphology. They retrospectively documented that children with otitis media (OM) present shorter anterior cranial base length and upper facial height than children without OM. They concluded that deviations in craniofacial growth and development under the influence of the occipital, sphenoid and temporal bones not only generate anomalies in the position of the ET but can also increase the tendency to contract OM [15]. As the ET is mechanically supported by the temporal bone, the latter might be one of the key structures involved in the pathogenesis of AOM.

Clinically, young children can present bony overlapping of the temporal, sphenoid and occiput bones causing restrictions in the malleability and mobility of the sutures between those bones. Such subtle bony misalignments, occurring while accommodating delivery or secondary to asymmetry in myofascial tension, are possible since a child’s cranial bones are surrounded by flexible membraneous and cartilaginous connective tissues [16]. Three or four days after birth, one can normally feel the edge to edge status of the temporal bone sutures by palpation assessment [17]. Persisting bone overlapping and suture restrictions involving the temporal bone are commonly assessed in the cranial osteopathic approach. As mentioned, those overlapping and restriction of sutures of the temporal bone may, to some extent, affect anatomical development or position of the ET and increase risk of AOM. Identification of suture restrictions of the temporal bone is clinically important since it can lead to creative and non-invasive treatment options, such as correction by cranial manipulations in young children before the age of ossification of the skull base. However, to our knowledge, no study has examined the relationship between suture restriction of the temporal bone and the development of AOM. This study thus investigates whether severe suture restriction of the temporal bone is a risk factor for the development of AOM in young children.

## Methods

### Design, setting and participants

A prospective cohort study followed 64 children 6 to 18 months of age and without prior history of AOM during the cold season (September 2009 to April 2010). Participants were recruited in community-based organizations offering recreational activities for families with young children in Sherbrooke (Québec, Canada) between May 2009 and September 2009. Children with congenital anomalies (specifically cleft palate or Down syndrome) and with hearing problems were excluded.

Parents who agreed to allow their child to participate signed an informed consent. The study protocol was approved by the Ethics Committee of the Centre Hospitalier Universitaire de Sherbrooke. Parents were instructed to continue standard care and consult their primary care physician when needed. No intervention was included in the study.

### Variables

Using a standardized questionnaire, a research assistant first collected data on baseline potential confounders and risk factors for AOM: a) specifically related to the child: gender, age (months), birth weight (grams), gestational age, use of pacifier, breastfeeding ≥ 6 months, history of upper respiratory tract infection (bronchiolitis, sinusitis and pneumonia diagnosed by a physician); and b) related to his/her environment: daycare attendance (≥3 days/week and for at least 3 months before the end of the study), presence of siblings (≥1), parental smoking and low socioeconomic status (<$30,000/year). Data on breastfeeding and daycare attendance were updated during the study. Following this questionnaire and blinded to its results, a qualified osteopathic practitioner, according to the World health organization Benchmarks for training in osteopathy, 2010 [18], assessed baseline temporal bone status (categorized as with or without severe suture restriction) using visual observation (noticeable displacement between the squamous and petrous parts or suture overlapping) and manual examination for each temporal bone.

**Figure 2:**
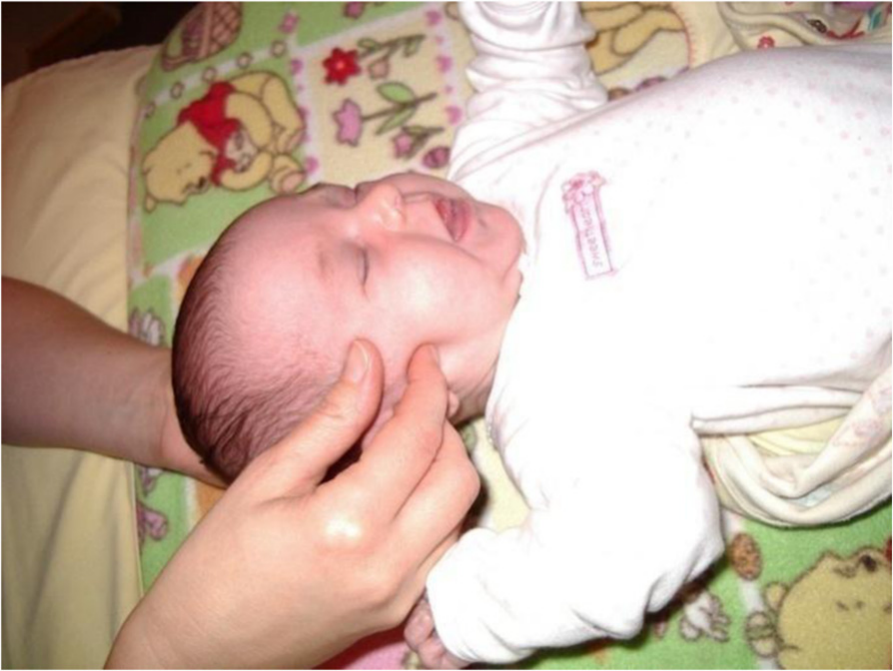
**Manual unilateral temporal bone mobility test.** To realized the mobility test, one hand is placed longitudinally under the occipital bone and the other hand uses a butterfly grip on the temporal bone (thumb and index finger bridging the zygomatic process while the ring and little fingers bridge the mastoid process).

Specifically, after bilateral observation of the temporal bone symmetry and general palpation of the shape of the head and cranial sutures [17], a manual unilateral temporal bone mobility test was used to identify restrictions that affect the temporal bone sutures (Figure 2). Using the occipital bone as a reference point, the temporal bone is mobilized on its arbitrary physiological axis (from the jugular surface to the petrous apex) in a motion called external rotation (to carry the superior border of the petrous portion anterolaterally towards the exterior of the skull) and, from the neutral position, in the opposite coupled motion (internal rotation). The mobility test assessed and compared the perception of compliance in one direction (external rotation) and in the opposite coupled motion (internal rotation). Temporal bone with severe suture restriction has no compliance in both directions, almost complete resistance in one direction (external or internal rotation), or visible displacement between the squamous and petrous parts of the temporal bone. On the other hand, in the absence of severe suture restriction of the temporal bone, compliance can be assessed in both directions with resistance occurring only near the end of the mobilization. Performed by an osteopathic practitioner in infants less than 6 months, interrater reliability of examination findings is 0.58 for the right temporal bone and 0.71 for the left temporal bone [19]. In a similar unpublished pilot study done as a part of the present study by Morin and Collette, the manual unilateral temporal bone mobility test presents good interrater reliability (Kappa 0.65) when used on children between 6 and 18 months old (n=22). Finally, due to their close anatomical relationship with the temporal bone and their mechanical influence on its mobility and development, positions of occipital and sphenoid bones were also considered. Overall position of the occipital bone in relation to the sphenoid bone (sphenobasilar synchondrosis) and symmetry of the condylar part of the occipital bone, located just behind the petrous part of the temporal bone, were estimated using classical manual cranial assessment described in osteopathic literature [20,21]. Manual assessment of cranial movement and occipital condylar parts position present an overall interrater reliability of 0.65 [19]. Temporal bone status was assessed only at baseline but events (head trauma) or manual interventions on cranial bones that might have affected temporal bone status were considered during the follow-up interview with parents described in the following section.

### Outcome

During the follow-up period (8 to 11 months), AOM had to be diagnosed by a physician (general practitioner, emergency room specialist or pediatrician) according to specific criterias currently included in guidelines of management of AOM. The diagnosis of AOM required 1) a history of acute onset, 2) presence of middle-ear effusion and 3) signs and symptoms of middle-ear inflammation [5]. The parent was instructed to present a reminder to document the presence of AOM, the side affected, the name of the physician and the date of consultation. Finally, a research assistant documented history of AOM in children using phone interviews with parents every 2 months. Physicians, parents and the research assistant were blinded to the child’s temporal bone status. Parent interviews are considered a good way to collect data on recent AOM outcomes in community-based studies where obtaining medical records is often impractical [22]. In fact, parental reporting of children’s recent AOM history has been shown to correlate well (kappa 0.88) with medical records [22]. Physicians were not involved in this study other than to diagnose the AOM and to fill out the reminder note.

### Sample size

Based on an alpha significance level of 5% and a power of 80%, a sample of 126 ears (63 children) was sufficient to detect a difference greater than 20% (effect size 0.45) between the incidence of AOM in ears with and without severe suture restriction of the temporal bones.

### Statistical analyses

Participants’ characteristics were described by means and standard deviations or frequencies and percentages according to the type of variable (numerical or categorical, respectively). T-tests, chi square and Fisher’s exact test were calculated with SPSS (version 17.0, Chicago, IL, USA) to compare children with or without severe suture restriction of the temporal bone. Hierarchical linear and nonlinear (multilevel) model was performed using HLM (version 6.0, Scientific Software International, Chicago, IL, USA) to investigate whether severe suture restriction of the temporal bone was a risk factor for AOM. Since our data has a hierarchical structure (two ears having specific characteristics nested into one child) a two-level model was used. The level one identified associations between AOM and ear-level variables and the level two considers the influence of the child-level variables. Potential confounders and potential risk factors for AOM were associated with either the ear (level one) or the child (level two). Using random effects to control for other risk factors not known or not included, the model was adjusted first with the independent variable (temporal bone status), age and gender. Second, characteristics specifically related to the child were added to the model and, finally, those related to the child’s environment. When used in cohort studies, hierarchical liner or non-linear model yields an odds ratio (OR) rather than a risk ratio (RR). However, when the outcome of interest is common in the study population (≥10%), such as AOM, the adjusted OR should be transformed to an estimate of the association that better represents the RR [23]. Odds ratios obtained by hierarchical linear or non-linear modeling in this study were thus transformed to RRs (95% CI) using Zhang and Yu’s formula [23].

## Results

Parents of 75 children eligible to participate in this study were approached, and 65 agreed. Loss in follow-up was limited to 1.5% since only one parent could not be reached to document history of AOM in his child. Results presented are thus based on a sample size of 64 children (128 ears).

Both groups, with and without severe suture restriction of the temporal bone, had similar baseline characteristics (Table 1). No pre-term or low birth weight children were found in the cohort. The children’s age ranged from 6 to 17 months at the time of inclusion and girls and boys were almost equally represented. More than one third of the children had at least one ear with severe suture restriction of the temporal bone, while almost one quarter of the ears presented such restriction (Table 2).

**Table 1:** Baseline characteristics of participants

**Characteristics**	**All**	**Without severe restriction**	**With severe restriction**	
	**(n = 64)**	**(n = 41)**	**(n = 23)**	
Continuous variables	Mean (SD)	Mean (SD)	Mean (SD)	*P* Value
Age, months	9.9 (3.1)	9.7 (3.1)	10.4 (3.1)	0.38 ^a^
Birth weight, g	3373 (554)	3410 (476)	3307 (677)	0.48 ^a^
Gestational age, weeks	39.6 (1.8)	40.0 (1.4)	39.0 (2.3)	0.07 ^a^
Categorical variables	Frequency (%)	Frequency (%)	Frequency (%)	*P* Value
Gender (Boy)	35 (54.7)	23 (56.1)	12 (52.2)	0.76 ^b^
Use of pacifier (Yes)	46 (71.9)	30 (73.2)	16 (69.6)	0.76 ^b^
Daycare attendance (Yes)	43 (67.2)	27 (65.9)	16 (69.6)	0.76 ^b^
Siblings (Yes)	28 (43.8)	19 (46.3)	9 (39.1)	0.58 ^b^
Low socioeconomic status (< $30,000/year)	13 (20.3)	8 (19.5)	5 (21.7)	1.00 ^c^
Breastfeeding ≥ 6 months (Yes)	43 (67.2)	29 (70.7)	14 (60.9)	0.42 ^b^
Parental smoking (Yes)	16 (25.0)	7 (17.1)	9 (39.1)	0.05 ^c^
History of URTI (Yes)	11 (17.2)	7 (17.1)	4 (17.4)	1.00 ^c^
Bronchiolitis	7 (10.9)	4 (9.8)	3 (13.0)	0.70 ^c^
Sinusitis	2 (3.1)	1 (2.4)	1 (4.3)	1.00 ^c^
Pneumonia	3 (4.7)	3 (7.1)	0 (0)	0.55 ^c^

SD: standard deviation, g: gram, URTI: upper respiratory tract infection.

^a^***P*** Value of the T Test. ^b^***P*** Value of the Chi-square Test. ^c^***P*** Value of the Exact Fischer Test.

**Table 2:** Distribution of severe suture restriction of the temporal bone and cranial base characteristics

**Children (n = 64)**	**Frequency**	**Percentage**
Severe suture restriction of the temporal bone	23	35.9
Asymmetry of occipital condylar parts (total)	30	46.9
Sphenobasilar synchondrosis perturbation	36	56.3
**Ears (n = 128)**		
Severe suture restriction of the temporal bone	29	22.7
Internal rotation of temporal bone	8	6.3
External rotation of temporal bone	18	14.1

At follow-up, 29 children (45%) were diagnosed with at least one AOM episode. An AOM episode was found in about half (14; 48.3%) of the ears associated with temporal bone previously identified as having severe suture restriction and in more than one quarter (28; 28.3%) of those without severe suture restriction.

Severe suture restriction of the temporal bone was identified as a significant risk factor for AOM, even when considering potential baseline confounders and other potential risk factors (Table 3). In the final model, higher risk of developing AOM was explained by severe suture restriction of the temporal bone, use of pacifier and younger age (Table 4).

**Table 3:** Results of the three hierarchical non-linear models of risk factors for AOM

**Model 1**			**Model 2**		**Model 3**	
**Variable**	**Relative Risk (95% CI)**	***P *Value**	**Relative Risk (95% CI)**	***P *Value**	**Relative Risk (95% CI)**	***P *Value**
**Severe suture restriction of the temporal bone (Yes)**	2.01 (1.25 to 2.69)*	**<0.01**	2.10 (1.41 to 2.83)*	**<0.01**	2.26 (1.43 to 2.91)*	**<0.01**
Internal rotation of the temporal bone (Yes)	1.22 (0.41 to 2.40)	0.68	0.86 (0.19 to 2.34)	0.81	0.84 (0.12 to 2.58)	0.83
External rotation of the temporal bone (Yes)	0.78 (0.31 to 1.64)	0.56	0.47 (0.15 to 1.24)	0.14	0.42 (0.13 to 1.14)	0.09
**Age**	0.29 (0.11 to 0.72)*	**<0.01**	0.25 (0.11 to 0.51)*	**0.001**	0.22 (0.10 to 0.52)*	**0.001**
Gender (Boy)	1.30 (0.65 to 2.12)	0.42	1.58 (0.84 to 2.39)	0.14	1.52 (0.82 to 2.30)	0.16
**Use of pacifier (Yes)**			2.47 (1.49 to 3.12)*	**<0.01**	2.59 (1.51 to 3.22)*	**<0.01**
History of upper respiratory tract infection (Yes)			0.34 (0.08 to 1.15)	0.09	0.36 (0.08 to 1.32)	0.14
Breastfeeding ≥ 6 months			0.77 (0.35 to 1.46)	0.45	0.73 (0.29 to 1.53)	0.45
Sphenobasilar synchondrosis perturbation (Yes)			1.56 (0.83 to 2.37)	0.15	1.64 (0.90 to 2.42)	0.09
Asymmetry of the occipital condylar parts (Yes)			0.97 (0.48 to 0.68)	0.93	1.01 (0.50 to 1.74)	0.98
Parental smoking (Yes)					0.66 (0.19 to 1.71)	0.44
Daycare (Yes)					1.73 (0.90 to 2.58)	0.09
Socioeconomic status (<$30,000 / year)					1.99 (0.93 to 2.90)	0.07

* denotes significance at < 0.01.

**Table 4:** Significant risk factors in the final explanatory model for AOM after hierarchical linear and nonlinear (multilevel) models (n = 64 children and 128 ears)

	**Relative Risk (95% CI)**	***P *Value**
Age (months)	0.22 (0.10 to 0.52)	0.001
Severe suture restriction of the temporal bone	2.26 (1.43 to 2.91)	<0.01
Use of pacifier	2.59 (1.51 to 3.22)	<0.01

## Discussion

### Principal findings

This prospective cohort study is the first to examine and demonstrate the role of severe suture restriction of the temporal bone as a risk factor for the development of AOM in infants aged from 6 months up to 30 months by the end of follow-up. Such empirical demonstration is consistent with current anatomical and physiological knowledge, and also allows furthers understanding of the possible mechanical influence of the temporal bone on ET and, consequently, AOM.

### Comparison with other studies

The results of the present study are consistent with previous studies that examined the influence of craniofacial morphology on the ET. DiFrancesco and colleagues (2008) retrospectively documented a relationship between shorter anterior cranial base length and OM using specific landmarks on digitalized standardized lateral head radiographs of 67 children with or without history of OM [15]. Our results add prospective evidence of the influence of restriction affecting sutures between the temporal bone and other bones of the cranial base and subsequent development of AOM. To our knowledge, no other prospective study has looked at this relationship.

### Possible explanations and implications

These prospective results on occurrence of AOM in ears previously associated with severe suture restriction of the temporal bone suggest that the temporal bone has an influence on ET positioning and function. In fact, the ET is anatomically influenced by bones of the cranial base since its bony portion is located within the temporal bone and its fibro-cartilaginous portion travels between the temporal and sphenoid bones [9]. Perturbations in the anatomical relationship of those structures can mechanically affect the isthmus, the smallest portion of the ET, which acts as a functional sphincter and has a diameter of only 0.5 mm in children [24]. It is not surprising that such a small diameter is sensitive to anatomical variations in the area. Indeed, normal ET function requires good positioning and mobility of all bony and myofascial neighboring structures [25]. A change of just a few degrees in the craniofacial morphology associated with suture restrictions, for example, of the temporal bone, could have significant consequences on ET function and play a role in the pathogenesis of AOM [15]. Fortunately, the influence of suture restriction of the cranial base bones on the development of AOM may be a modifiable risk factor, especially in young children. While the sphenoid fontanel is usually closed by 2 years of age, a high resolution computed tomography (CT) study revealed that closure of the synchondrosis between occipital and sphenoid bones by ossification occurs only at 13 years of age [26]. Furthermore, the petrous part of the temporal bone and the basiocciput remain apart during the entire life, separated by vestiges of cartilaginous synchondrosis [16,27]. Akin to the use of a pacifier after 6 months of age [28], severe suture restriction of the temporal bone in the young child, whose skull is still very malleable, could be a modifiable factor. Such suture restriction is an interesting target for creative and non-invasive interventions aimed at optimizing ET position and function through its bony environment. Clinically, these results support the need to explore new treatments based on modern insights into the pathophysiology of otitis media [1]. Indeed, the results suggest a holistic vision of ET dysfunction, based not only on the tube itself but also on its surroundings and connections. Specifically, the association between the presence of severe suture restriction of the temporal bone and AOM suggests that temporal bone sutures mobility is important for normal growth and maturation of the ET. Suture restriction of other structures surrounding the ET such as the occiput, sphenoid or facial bones might also affect its maturation. Suture restriction of the temporal bone is an interesting unilateral risk factor that might be considered when facing unilateral recurrent AOM. Future studies should investigate the screening of children at risk for AOM or having recurrent AOM, especially unilateral, using a cranial mobility test: a simple, non-invasive, side-effect-free assessment. Subsequently, the efficacy of manual cranial interventions in young children to reduce occurrence of AOM [29,30] needs to be further studied in order to explore this mechanical avenue to prevent AOM.

### Strengths and weaknesses of the study

The strengths of the study include a population based longitudinal design, consideration of many important variables (including modifiable risk factors for AOM) and rigorous methodology, including validated procedures and assessments [17,19]. Since the suture restriction of the temporal bone was asses prior to the occurrence of AOM, the prospective design of the study reduces the selection bias. The study was conducted using a previously demonstrated successful procedure (rigorous and regular follow-up calls every 2 months) [22,31] in addition to the reminder note to be completed by the treating physician at the time of diagnosis of AOM, which help to limit recall bias and the dropout rate. Furthermore, as the reminder note specifically inquired about the side of the AOM and the exact diagnosis, this procedure also avoided the need to interpret medical records and asked treating physicians to provide a precise diagnosis. Loss in follow-up, the most important bias to consider in cohort study, was limited to 1.5%. This study also has some limitations. First, despite the availability of clear guidelines [5,32], variability in the diagnosis of AOM by physicians and pediatricians is possible. Second, this research project may have encouraged parents to consult about AOM, which they would not otherwise have done. However, as both treating physicians and parents were blinded to temporal bone status, under- and over-diagnosis of AOM should be equally distributed in both groups. Such potential non-differential diagnosis misclassification increased the similitude between the exposed and non-exposed groups and might leads to underestimate the real association between suture restriction of the temporal bone and AOM [33]. Finally, the initial and single assessment of the temporal bone status did not allow for monitoring of potential changes in bone status that might have happened during the study. Such changes were nevertheless considered during the follow-up parent interviews, and no children received a cranial intervention before the occurrence of the first AOM and only one child consulted for trauma to the head. This child had his first episode of AOM one month later, and was considered in the analysis as not having severe suture restriction of the temporal bone (result of his initial assessment). Generalization of the study results has to be conformed and validated in other settings and populations.

## Conclusion

This study prospectively, clinically and statistically demonstrates the association between the presence of severe suture restriction of the temporal bone and the development of AOM in young children. This is the first time that severe suture restriction of the temporal bone has been empirically identified as a risk factor for acute otitis media. Subsequent studies are needed to determine if this mechanical risk factor can be modified.

### Patient consent form

Parents of identifiable participant have provided their consent to publication.

## Abbreviations

ET: Eustachian tube; AOM: Acute otitis media; RR: Relative Risk; OM: Otitis media; OR: Odd Ratio.

## Competing interest

The authors declare that they have no competing interests.

## Authors’ contributions

CM, DD, JMM and ML for conception and design of the study, analysis and interpretation of data, critical revision of the manuscript for important intellectual content, and administrative, technical and material support. CM for acquisition of data, CM and DD for drafting of the manuscript, statistical analysis and obtaining funding. ML for statistical analysis. All authors read and approved the final manuscript.

## Pre-publication history

The pre-publication history for this paper can be accessed here:

http://www.biomedcentral.com/1471-2431/12/181/prepub
